# Cross-Cultural Differences and Psychometric Properties of the Japanese Actions and Feelings Questionnaire (J-AFQ)

**DOI:** 10.3389/fpsyg.2021.722108

**Published:** 2021-08-20

**Authors:** Charlotte F. Huggins, Isobel M. Cameron, Neil W. Scott, Justin H. G. Williams, Sakiko Yoshikawa, Wataru Sato

**Affiliations:** ^1^Centre for Genomic and Experimental Medicine, Institute of Genetics and Cancer, University of Edinburgh, Edinburgh, United Kingdom; ^2^School of Medicine, Medical Sciences and Nutrition, Foresterhill, University of Aberdeen, Aberdeen, United Kingdom; ^3^Child and Youth Mental Health Service, Gold Coast Health, Strathpine, QLD, Australia; ^4^Kokoro Research Centre, Kyoto University, Kyoto, Japan; ^5^Psychological Process Team, BZP, Robotics Project, RIKEN, Saitama, Japan

**Keywords:** actions and feelings questionnaire, cross-cultural differences, motor empathy, translation, psychometrics, differential item functioning

## Abstract

**Aims:** We aimed to assess the psychometric properties of a Japanese version of the Actions and Feelings Questionnaire (J-AFQ), an 18-item self-report measure of non-verbal emotional communication, as well as to examine its transcultural properties.

**Methods:** The J-AFQ was administered to 500 Japanese adults (age 20–49, 250 male), alongside the Japanese Broad Autism Phenotype Questionnaire (BAPQ-J) and Empathy Quotient (EQ-J). These were compared to a group of 597 British and Irish participants (age 16–18, 148 male). J-AFQ was assessed in terms of validity by confirmatory factor analysis and convergence with BAPQ-J and EQ-J using Pearson correlation. Internal consistency and differential item functioning (DIF) were assessed and compared between Japanese and UK/Irish participants.

**Results:** Reversed worded items (RWIs) showed poor item-total correlations but excluding these left a 13-item version of the J-AFQ with good internal consistency and content validity. Consistent with the English version, J-AFQ scores correlated with EQ and lower BAPQ scores. However, comparing across cultures, J-AFQ scores were significantly lower in the Japanese sample, and there was evidence of important DIF by country in over half of the J-AFQ items

**Conclusion:** Cultural differences in attitudes to self-report, as well as increased acquiescence to RWI's also seen in previous studies, limit the value of the 18-item instrument in Japanese culture. However, the 13-item J-AFQ is a valid and reliable measure of motor empathy, which, alongside the English version, offers promise for research in motor cognition and non-verbal emotional communication across cultures.

## Introduction

Understanding other people's non-verbal communication, such as through facial expression and gesture, is fundamental to social interaction. Successful social communication requires both agents to effectively express their internal states through bodily and facial movements, as well as appropriately interpret the gestures of other people. This ability, sometimes termed “motor empathy” (Blair, 2005), is a key component of empathy (Decety and Meyer, 2008).

Facial and bodily action may influence how emotions are experienced. For instance, stimulating the muscles frequently involved in various emotional expressions increases the intensity of the experience of that emotion, even if the stimulation is unrelated (Strack et al., 1988; Lewis, 2012; Mori and Mori, 2013). Moreover, mimicry of other's facial and bodily actions may simulate similar emotional states in the self, facilitating the ability to identify and understand other people's emotional states (Van der Graaff et al., 2016). As such, individual differences in motor empathy and non-verbal communication may be important in emotion and socialization.

Furthermore, impaired non-verbal communication is an important diagnostic feature of autism, central to instruments such as the Autism Diagnostic Observation Schedule (Lord et al., 2000) and the Autism Diagnostic Interview (Rutter et al., 2003). Children on the autism spectrum also demonstrate abnormalities of imitation, a key component of motor empathy (Williams et al., 2004). As such, motor empathy may be a valuable target for intervention and study in autism.

Despite this, motor empathy has received relatively little attention in research compared to both cognitive and emotional empathy. One key reason for this is that motor empathy is often measured through neuroimaging (e.g., Schulte-Rüther et al., 2017), facial electromyography (e.g., Van der Graaff et al., 2016), or facial action coding (e.g., Khvatskaya and Lenzenweger, 2016). These methods are time-consuming and expensive for researchers, burdensome for participants, and infeasible for large samples of child or clinical populations.

Furthermore, motor empathy is often not explicitly measured in common self-report measures of empathy such as the Interpersonal Reactivity Index (IRI; Davis, 1980) or the Empathy Quotient (EQ; Baron-Cohen and Wheelwright, 2004), and there are few methods suitable to measure motor empathy quickly in the clinic and research. Williams et al. (2016) noted that few, if any, self-report measures of motor empathy have been validated for use, making it difficult to assess how individual differences in motor empathy contribute to socioemotional outcomes.

To address this gap in the literature, Williams et al. (2016) developed the Actions and Feelings Questionnaire (AFQ). This is an 18-item self-report measure, quantifying motor cognition and empathy in adults. The questionnaire showed good internal consistency and test-retest reliability, as well as high convergent validity with the EQ. Moreover, higher scores on the AFQ were associated with greater activity on the somatosensory cortex during imitation (Williams et al., 2016), consistent with the hypothesis that the AFQ indexes emotional action-awareness. Female participants also had significantly higher AFQ scores than male participants (Williams et al., 2016), falling in line with research on empathy. AFQ scores were also lower in adults on the autism spectrum (Williams and Cameron, 2017), and greater AFQ scores were associated with autistic traits in typical populations (Huggins et al., 2019). These findings suggest that the AFQ may be an effective measure of motor cognition and empathy, as well as a useful screening tool for autism.

A key limitation of the AFQ is that it has been implemented in largely Western populations, although a recent study has validated it for use in Dutch (Van der Meer et al., 2021). Yet non-verbal emotional communication may vary culturally in terms of its reliance upon non-verbal communication. Our current study aimed to assess the psychometric properties of a translation of the AFQ for use in Japanese samples, as well as examine whether they differed from a British sample.

Cultures vary in their “display rules,” reflecting the extent to which it is appropriate to express one's own emotions both verbally and non-verbally. Japanese display rules tend to discourage more intense emotional expression compared to Western cultures (Matsumoto, 1990; Matsumoto et al., 2008). Moreover, emotional communication in Japan may be less direct than that in most Western cultures, as evidenced by Japanese participants reporting it being less appropriate to express intense emotions compared to Western participants (Safdar et al., 2009). It has been suggested that emotions in Japan tend to be expressed in more subtle ways than in Western cultures (Yoshie and Sauter, 2020).

Japanese emotional cues may be more subtle and context-specific than Western cues. For instance, Japanese participants tend to be more attentive to contextual cues when decoding emotional expressions of others (Masuda and Nisbett, 2001), and also tend to pay more attention to vocal tone over both facial cues (Tanaka et al., 2010) and verbal content (Ishii et al., 2003). Finally, incongruence between bodily and facial cues of emotion are more disruptive to emotional recognition for Japanese compared to Western participants (Bjornsdottir et al., 2017). Thus, while “reading the room” is a valuable skill in any culture, it may be particularly important in Japan, where emotion cues may be more subtle.

It has been suggested that the greater cultural focus on “reading the air” throughout development accounts for differences in neural activation between Japanese and Western populations during “Theory of Mind” (ToM) tasks. Koelkebeck et al. (2011) compared Western and Japanese participants on the Moving Shapes task (Abell et al., 2000). In this task, participants watched videos of colored triangles moving around a screen. There were three conditions—random, in which triangles made completely random movements; goal-directed, in which triangles made movements which were related to one another but did not indicate any degree of mind-reading; or TOM, in which triangles made movements indicative of relating to one another's “mental state.” Participants verbally described triangle movements while undergoing fMRI. It consequently emerged that while verbal descriptions did not differ between cultures, Japanese participants showed lower medial pre-frontal cortex activation during the ToM condition compared to the Western group. The authors argued that this was due to Japanese populations having more practice reading non-verbal emotion cues, and thus need to devote fewer cognitive and neural resources to such a task (Koelkebeck et al., 2011). They suggested that Japanese populations may be more sensitive to non-verbal emotional communication than Western populations.

Moreover, “*omoiyari*”—the ability to understand the unexpressed feelings of others (Lebra, 1976) and sometimes translated as “empathy”—is one of the traits most frequently chosen by Japanese young people are asked to describe their “ideal self” (Shimizu, 2000). This suggests that while being adept at reading the non-verbal emotional cues of others is a socially desirable trait in almost any culture, it is particularly valued in Japan.

The primary goal of our study was to develop and validate a Japanese version of the Actions and Feelings Questionnaire (J-AFQ) for use in Japanese populations. As few self-report measures of motor empathy exist in the literature, this may provide a useful tool for screening and research in Japanese samples. To assess the psychometric properties of J-AFQ, we analyzed internal consistency and conducted a confirmatory factor analysis. We additionally assessed participants' empathic and autistic traits and tested the convergent validity of J-AFQ. Our secondary goal was to examine whether there are cultural differences in AFQ individual question responses and overall item scores. We predicted that AFQ scores would be higher in the Japanese compared to the Western sample, due to the greater salience of non-verbal emotional communication abilities in Japan.

## Methods

### Participants

500 Japanese adults (250 male, 250 female) were recruited online through the survey company Rakuten Insight. Rakuten Insight advertised the study in Japanese on their website, which was accessible to any internet user. Registered Rakuten Insight users were also invited by email. Ages ranged from 20 to 49, with a median age of 35 (interquartile range = 14).

A comparison group of adults from the UK and Ireland were sampled from a previous study of the AFQ (Williams and Cameron, 2017). Social networking and e-mail lists were used to circulate a link to the questionnaire, administered through SurveyMonkey. After excluding participants on the autism spectrum and those from outside of the UK and Ireland, this provided 597 participants (148 male, 449 female) for comparison. Ages ranged from 16 to 88, with a mean age of 42.21 (SD = 14.95).

### Actions and Feelings Questionnaire

The Actions and Feelings Questionnaire is an 18-item self-report questionnaire intended to measure motor cognition and empathy. Higher scores indicate higher levels of motor empathy, reflected through greater sensitivity to the emotion-related actions of others, as well as a stronger tendency toward using motor imagery and expressing emotion through motor action.

Participants respond to each question on a four-point Likert scale, reflecting “Strongly Disagree” to “Strongly Agree.” These responses are coded from 0 to 3, respectively. Five items are negatively scored. Scores across all items are summed to produce total scores.

The English-language version has high internal coherence and test-retest reliability, is strongly correlated with Empathy Quotient (EQ; Baron-Cohen and Wheelwright, 2004), and scores are significantly higher in female populations (Williams et al., 2016). Within Western samples, it has a three-factor structure composed of the subscales “Feelings,” “Imagery” and “Animation” (Williams and Cameron, 2017). To create the J-AFQ (see Supplementary Material), AFQ was translated from English to Japanese by WS, a Japanese native speaker fluent in English. Items were then back-translated by both Japanese and English native speakers to ensure meaning was preserved.

### Empathy

Empathy was also measured with the Japanese version of the 15-item EQ (Baron-Cohen, 2005; Muncer and Ling, 2006). Seven items are reverse-scored. Scores are summed to produce totals, and higher scores reflect greater empathy.

The 15-item EQ has three subscales: “Cognitive,” “Emotional Reactivity,” and “Social Skills.” “Cognitive” measures cognitive empathy, such as the ability to predict and understand the feelings of others, measured through items such as “I can easily work out what another person might want to talk about.” “Emotional Reactivity” measures the tendency to react emotionally to others, through items such as “I really enjoy caring for other people.” “Social Skills” reflects skill and comfort in social situations, measured through items such as “I do not tend to find social situations confusing.”

### Autistic Traits

Autistic traits were measured with the Japanese version of the Broad Autism Phenotype Questionnaire (BAPQ-J; Sakai et al., 2014), a 36-item self-report questionnaire intended to assess autistic traits in neurotypical populations. 15 items are reverse-scored. The mean of items is calculated to produce the total, with higher scores reflecting greater autistic traits.

The BAPQ has three subscales, measuring “Aloofness,” “Pragmatic Language Skill” and “Rigidity.” “Aloofness” reflects disinterest in social situations and relationships with others, measured through items such as “I would rather talk to people to get information than to socialize.” “Pragmatic” reflects difficulties with social conversation and language, measured through items such as “I find it hard to get my words out smoothly.” “Rigidity” reflects rigid adherence to routine and inflexibility in habits, measured through items such as “I have a strong need for sameness from day to day.”

## Statistical Analysis

### Internal Consistency

Internal consistency of the J-AFQ was assessed with Cronbach alpha (acceptable value > 0.7; Cortina, 1993). These were computed for the total scale and the three subscales: “feelings,” “animation,” and “imagery.” Item-total correlations were calculated (acceptable values > 0.3; Everitt, 2002).

### Confirmatory Factor Analysis

Confirmatory factor analysis (CFA) was conducted to assess whether the Japanese data were a good fit to the UK-derived three-factor model (Williams and Cameron, 2017). Additionally, any revised model (following conduct of the internal consistency analysis) was also assessed. Where data were normally distributed, CFA with maximum likelihood (ML) estimation was conducted using IBM SPSS AMOS 25.

The following fit indices were computed: the comparative fit index (CFI) (values ≥ 0.95 are considered a good fit; Hu and Bentler, 1999); the root mean square error of approximation (RMSEA) (an acceptable fitting model value ≤ 0.07; Steiger, 2007); and the standardized root mean square residual (SRMR) (values <0.08 indicate an acceptable fit; Hu and Bentler, 1999). As with the UK AFQ (Williams and Cameron, 2017), we hypothesized likely correlated error between three pairs of items (Q11 and Q17, Q5 and Q9, Q14 and Q15). Items can be seen in Table 1.

**Table 1:** Item Total Correlations for the 18-item and 13-item AFQ.

		**Corrected item-total correlations**	
		**18-item AFQ**	**13-item AFQ**
1	I tend to pick up on people's body language	0.447	0.509
**2**	**To understand someone I rely on their words rather than their expression or gesture** ^**a**^	**−0.210**	**–**
3	To make sense of what someone else is doing, I might copy their actions	0.437	0.510
4	Music that I like makes me want to dance	0.483	0.513
5	In my mind's eye, I often see myself doing things	0.447	0.481
6	If talking on the phone, I am sensitive to someone's feelings by the tone of their voice	0.399	0.418
7	If others are dancing I want to join in	0.463	0.500
**8**	**My body movements do not tend to reflect the way I feel** ^**a**^	**−0.295**	**–**
9	I often imagine myself performing common actions	0.512	0.541
10	I would consider myself to be a “touchy-feely” person	0.440	0.482
**11**	**When I recall what someone said to me, I have to think hard to remember their facial expression at the time** ^**a**^	**−0.038**	–
12	I rely on seeing how a person looks me in the eye to gauge what they really feel	0.365	0.467
**13**	**I wouldn't tend to know what someone was feeling if they did not say** ^**a**^	**−0.202**	–
14	I move my hands a lot when I speak	0.370	0.437
15	I get animated when I am enthusiastic in conversation	0.488	0.527
16	I can easily bring to mind the look on someone's face when I remember telling them something	0.476	0.496
17	Acting things out helps me to understand them	0.457	0.519
**18**	**Watching somebody's body language is not a good way to judge their feelings** ^**a**^	**−0.166**	–

*Items with values <0.3 in bold*.

a *Reversed item*.

### Convergent Validity

Pearson correlation coefficients were computed for the J-AFQ with the EQ and AFQ-J with the BAPQ. It was hypothesized that motor cognition would be strongly associated with empathic attitude and inversely related to BAPQ scores.

### Cross-Cultural Comparisons

Japanese data were compared to a previously collected sample of participants from the UK and Ireland. One-Way ANOVA was used to cheque for differences in age between Japanese and Western participants. To compare cultural groups while also accounting for differences in age and gender, 2 (Cultural group: Western vs. Japanese) × 2 (Gender: male vs. female) two-way ANCOVAs were conducted, controlling for age as a covariate.

### Differential Item Functioning

Translating the AFQ raises the risk that items in the scale may change meaning due to linguistic or cultural factors. Differential item functioning (DIF) occurs when different groups respond differently to a particular item within a questionnaire subscale, even after accounting for their overall scores in that subscale (Scott et al., 2010). This can help identify individual scale items that are problematic or are not answered in the same way by different populations. Ordinal logistic regression DIF analyzes were conducted using Stata version 15 and compared the present Japanese sample with the UK/Ireland sample of Williams and Cameron (2017) (*n* = 597). Results are expressed as log odds ratios where negative values mean that the Japanese sample were more likely to endorse the item compared with the Western sample. It is important to consider the size of the DIF effect as well as the statistical significance. In this study questionnaire items with log odds ratios >0.64 or < −0.64 with *p*-value <0.001 were considered evidence of important DIF (Zieky, 1993). DIF analyzes were also controlled for age and sex.

## Results

Western participants (M = 42.21, SD = 14.95) were significantly older than Japanese (M = 35.18, SD = 8.09) participants, *F*_(1, 1100)_ = 88.89, *p* < 0.001. Chi-square tests likewise found significant differences between gender distribution in each group, X^2^(1) = 74.802, *p* < 0.001, with the Western sample having a higher proportion of female participants compared to the Japanese sample.

### Internal Consistency

Cronbach alpha was 0.687 for the total score, 0.151 for “Feelings,” 0.724 for “Animation,” and 0.673 for “Imagery.” Item-total correlations are shown in Table 1. Five items had values <0.3 and all belonged to the Feelings sub-scale. On the removal of these 5 items, Cronbach's alpha was 0.842 for the total score and 0.688 for Feelings.

The items with values below 0.3 were the five reverse-scored items. To cheque for coding errors, all data were re-coded from scratch twice by CFH on two separate occasions, once by hand and once through a custom MATLAB script. The same pattern emerged with each coding.

To confirm that the issue did not emerge in translation, a professional Japanese-to-English translator with no prior knowledge of the questionnaire or study back-translated the reversed items. No significant issues emerged in back-translation (full details of back-translation can be seen in Supplementary Material). Based on this, it was concluded that the item-total correlations were not the result of statistical error or confusion in translation and instead reflected real values of the J-AFQ data. Following this, analyzes were conducted on both the 13-item and 18-item J-AFQ.

### Confirmatory Factor Analysis

**Figure 1:**
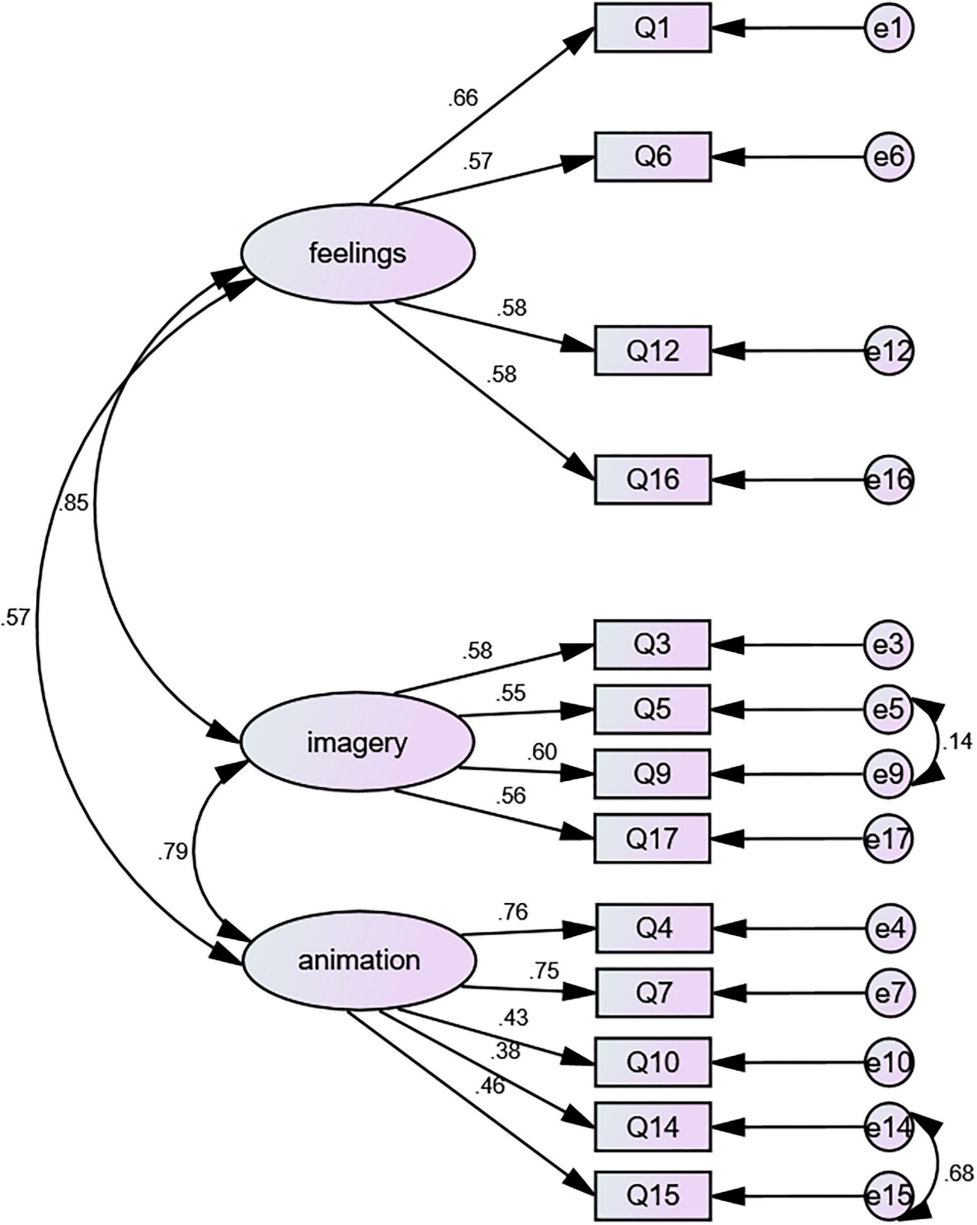
Graphical representation of the 13-item Japanese Actions and Feelings Questionnaire (AFQ) 3 factor model with correlated error.

Table 2 shows the fit indices for the J-AFQ. Model 1 considers the UK-derived 18 item 3-factor model. As noted, the 5 items with poor item-total correlations (see bold items in Table 1) were all items that required reverse scoring. In order to consider a systematic item-reversing bias, we hypothesized Model 2, which included a reversed item factor relating to these 5 items. The factor structure was also assessed in Model 3 where these 5 items were removed. The J-AFQ with 13 items generates a better fitting model than the 18-item J-AFQ models (with or without a reversed item bias factor). However, in common with the UK version, none of the J-AFQ models quite reach the stringent ≥0.95 level for CFI. The SRMR level has the best fit with the 13-item J-AFQ model. The RMSEA levels are not acceptable. However, the 90% CIs of Model 2 and 3 have an acceptable lower level. Considering the fit indices and the internal consistency statistics, we identify Model 3 as the best fitting model. Figure 1 presents a graphic illustration of the 13-item J-AFQ (Model 3).

**Table 2:** Fit Indices for Japanese 3-factor model with correlated error permitted. UK sample (Williams and Cameron, 2017) shown for comparison.

**3 factor model with permitted correlated error**	**X^**2**^**	**df**	***p*-value**	**CFI^**a**^**	**SRMR^**b**^**	**RMSEA^**c**^**	**90%CI^**d**^**
Japanese (18 item) Japanese (18 item) reverse Japanese (13 item)	571 462 248	129 124 60	<0.001 <0.001 <0.001	0.813 0.857 0.900	**0.0788** **0.0716** **0.0711**	0.083 **0.074** 0.079	0.076-0.090 0.067-0.081 0.069-0.090
UK	866	129	<0.001	0.916	**0.0721**	**0.064**	0.060-0.068

a *Comparative fit index*;

b *Standardized root mean square residual*;

c *Root mean square error of approximation*;

d *Confidence intervals. Indices in bold met criteria for acceptable fit*.

### Convergent Validity

Pearson's correlation co-efficients were calculated to examine the relationship between both versions (18-item and 13-item) of the J-AFQ with EQ and BAPQ total scores. The 18-item AFQ was significantly correlated with both EQ scores, *r*_(498)_ = 0.364, *p* < 0.001, and BAPQ scores, *r*_(498)_ = −0.184, *p* < 0.001. The 13-item AFQ was also significantly correlated with EQ, *r*_(498)_ = 0.284, *p* < 0.001, and BAPQ, *r*_(498)_ = −0.122, *p* = 0.006. Although relationships were significant in the expected directions, they were smaller in magnitude compared to previous Western samples (Williams and Cameron, 2017).

### Cultural Group Comparisons

To compare Total-AFQ scores by both gender and culture, two-way ANCOVAs were conducted, controlling for age as a covariate. See Table 3 for averages by cultural group and gender. Means reported in the text for this analysis are estimated marginal means and standard errors.

**Table 3:** Mean and standard deviations of AFQ scores by cultural group and gender.

	**Japan (** ***N*** **=** **500)**			**UK and Ireland (** ***N*** **=** **597)**		
	**Male**	**Female**	**Total**	**Male**	**Female**	**Total**
	**(*n* = 250)**	**(*n* = 250)**		**(*n* = 148)**	**(*n* = 449)**	
**AFQ Total [18 Item]**	25.58 (6.25)	26.06 (5.85)	25.82 (6.05)	29.32 (7.83)	34.69 (6.38)	33.36 (7.15)
Feelings	14.12 (2.67)	14.36 (2.50)	14.24 (2.58)	16.57 (4.36)	18.92 (3.63)	18.34 (3.96)
Imagery	5.13 (2.47)	4.74 (2.45)	4.93 (2.460)	5.09 (2.62)	5.61 (2.54)	5.48 (2.57)
Animation	6.33 (3.07)	6.97 (3.15)	6.65 (3.12)	7.67 (3.03)	10.17 (2.79)	9.55 (3.04)
**AFQ Total [13 Item]**	17.44 (6.73)	17.72 (6.54)	17.58 (6.63)	20.25 (6.24)	24.37 (5.78)	23.35 (5.78)
Feelings	5.99 (2.38)	6.01 (2.35)	6.00 (2.36)	7.49 (2.20)	8.59 (1.83)	8.2 (1.98)

For 18-item Total-AFQ scores, a significant main effect of culture was found, *F*_(1, 1092)_ = 259.653, *p* < 0.001. Western participants (M = 32.42, SE = 0.31) scoring significantly higher than Japanese participants (M = 25.45, SE = 0.29). A significant main effect of gender also emerged, *F*_(1, 1092)_ = 42.344, *p* < 0.001, with women (M = 30.29, SE = 0.25) scoring higher than men (M = 27.58, SE = 0.33). A significant interaction between Culture x Gender was found, *F*_(1, 1092)_ = 30.188, *p* < 0.001. Western women (M = 34.91, SE = 0.301) scored significantly higher than Western men (M = 29.93, SD = 0.53), *F*_(1, 1092)_ = 67.742, *p* < 0.001. However, scores between Japanese women (M = 25.66, SE = 0.41) and Japanese men (M = 25.24, SE = 0.41) did not significantly differ, *F*_(1, 1092)_ = 0.559, *p* = 0.455.

ANCOVAs were repeated for the 13-item Total AFQ scores. Again, Western participants (M = 22.72, SE = 0.29) scored significantly higher than Japanese participants (M = 17.21, SE = 0.27), *F*_(1, 1092)_ = 184.836, *p* < 0.001. Women (M = 20.95, SE = 0.24) also scored significantly higher than men (M = 18.98, SE = 0.31), *F*_(1, 1092)_ = 25.740, *p* < 0.001. A significant interaction between Culture x Gender was found, *F*_(1, 1092)_ = 20.639, p < 0.001. Western women (M = 24.58, SE = 0.28) scored significantly higher than Western men (M = 20.85, SE = 0.50), *F*_(1, 1092)_ = 42.490, *p* < 0.001. However, no significant differences emerged between Japanese women (M = 17.32, SE = 0.38) and Japanese men (M = 17.11, SE = 0.38), *F*_(1, 1092)_ = 0.155, *p* = 0.694.

On both 18-item and 13-item Total AFQ scores, Japanese participants scored significantly lower than UK and Ireland populations. Also, while gender differences emerged clearly in the Western populations, no gender differences emerged among the Japanese participants. Comparisons were also conducted to consider cultural groups by subscale scores.

A main effect of culture was found on every subscale, with Japanese participants scoring significantly lower than Western controls (Feelings-18: *F*_(1, 1092)_ = 246.70, *p* < 0.001; Imagery: *F*_(1, 1092)_ = 22.71, *p* < 0.001; Animation. *F*_(1, 1092)_ = 173.11, *p* < 0.001; Feelings-13: *F*_(1, 1092)_ = 204.83, *p* < 0.001).

A main effect of gender was found on every subscale apart from “Imagery,” with women scoring higher on both 18-item and 13-item “Feelings” subscales (18-item: *F*_(1, 1092)_ = 34.68, *p* < 0.001; 13-item: *F*_(1, 1092)_ = 15.35, p < 0.001), as well as Animation subscale, *F*_(1, 1092)_ = 15.348, *p* < 0.001.

Finally, a significant interaction was found between Culture x Gender for every subscale. Western women scores higher on the full Feelings scale, *F*_(1, 1092)_ = 53.019, *p* < 0.001), the short Feelings subscale, *F*_(1, 1092)_ = 28.035, *p* < 0.001), and the Animation subscale, *F*_(1, 1092)_ = 69.527, *p* < 0.001). No gender differences for Western participants on the Imagery subscale emerged, *F*_(1, 1092)_ = 2.002, *p* = 0.157).

No significant differences emerged by gender for Japanese participants on either Feeling subscales or the Imagery subscale (Feelings-18: *F*_(1, 1092)_ = 0.612, *p* = 0.434; Imagery: *F*_(1, 1092)_ = 3.767, *p* = 0.053); Feelings-13: *F*_(1, 1092)_ = 0.008, *p* = 0.930). However, Japanese women (M = 6.791, SE = 0.187) scored significantly higher than Japanese men (M = 6.173, SE = 0.187) on the Animation subscale, *F*_(1, 1092)_ = 5.575, *p* = 0.018.

### Differential Item Functioning

DIF analyzes were conducted for each item in every subscale, see Table 4 for full DIF analyzes.

**Table 4:** Differential Item Functioning for items on each subscale, controlling for total scores, age, and gender.

	**Log odds ratio**	***P-value***
**1. 4-items Feeling subscale**		
AFQ1	**2.486**	**<0.001**
AFQ6	0.830	0.601
AFQ12	**−1.194**	**<0.001**
AFQ16	**−1.017**	**<0.001**
**2. 9-item Feelings subscale**,		
AFQ1	**2.183**	**<0.001**
AFQ2	–0.539	<0.001
AFQ6	0.318	0.033
AFQ8	**−0.934**	**<0.001**
AFQ11	**−0.651**	**<0.001**
AFQ12	–0.356	0.011
AFQ13	0.573	<0.001
AFQ16	0.573	<0.001
AFQ18	**−0.802**	**<0.001**
**3. Imagery subscale**		
AFQ3	–0.166	0.211
AFQ5	**1.297**	**<0.001**
AFQ9	**−0.660**	**<0.001**
AFQ17	**−0.647**	**<0.001**
**4. Animation subscale**		
AFQ4	**0.662**	**<0.001**
AFQ7	0.509	<0.001
AFQ10	**−1.051**	**<0.001**
AFQ14	0.618	<0.001
AFQ15	0.570	<0.001

*Bolded items indicate p < 0.001 and log OR > 0.64 or log OR < −0.64*.

In the four-item DIF analyzes for the 4-item Feelings subscale, Western participants were more likely to endorse AFQ1, whereas Japanese participants were more likely to endorse AFQ12 and AFQ16, relative to other items in the scale. In the analysis of the 9-item Feelings subscale, Western participants were more likely to endorse AFQ1, whereas Japanese participants were more likely to endorse AFQ8, AFQ11, and AFQ18. These findings suggest large, statistically significant DIF effects by nation for both versions of this subscale.

For the 4-item Imagery subscale, Western participants were significantly more likely to endorse AFQ5, whereas Japanese participants were more likely to endorse AFQ9 and AFQ17.

In the 5-item Animation subscale, controlling for total subscale score, age, and gender, Western participants were more likely to endorse AFQ4, whereas Japanese participants were more likely to endorse AFQ10.

The results indicate statistically significant DIF effects for most items, suggesting many items are answered differently by the Japanese sample even after controlling for the total score in that subscale and adjusting for age and gender. Over half the items are associated with a log odds ratio with a magnitude >0.64, suggesting practically important DIF. These analyses do not, however, determine whether DIF effects are associated with the translation or with cultural factors.

## Discussion

We aimed to develop and validate a Japanese translation of the AFQ, J-AFQ, for use in the general population, as well as examine the transcultural properties of this measure. We initially found that the five reversed items on the AFQ had poor item-total correlations within a Japanese sample and that this was unlikely to be attributed to translation differences. Excluding these items leaves us with a 13-item measure with good internal consistency and satisfactory convergent validity.

As such, we conclude that the J-AFQ is a valid way to measure motor empathy in this context. Examining transcultural properties, we found that, against expectations, Japanese participants had significantly lower scores on the AFQ compared to a Western sample. Moreover, we found significant differences in Differential Item Functioning, suggesting Japanese participants respond to some AFQ items in a qualitatively different manner than UK and Ireland participants. Finally, unlike in Western samples, no gender differences emerged on AFQ scores among Japanese participants.

A key issue in terms of validity is that reversed items showed poor item-total correlations in the Japanese sample, and this was unlikely to be due to a coding error. Wider findings suggest that reverse coding is a common cause of difficulty within cross-cultural research. On a consumer research questionnaire, reverse-worded items (RWI) and positively-worded items (PWI) significantly correlated within American samples, but did not correlate with one another for Japanese participants, and also showed weaker inter-item correlations (Wong et al., 2003). This was suggested to be due to a greater tendency toward acquiescence in Japanese participants, as participants from collectivist cultures, such as Japan, show greater acquiescence in survey-taking compared to those from individualist cultures (Johnson et al., 2005).

Reversed-worded items causing difficulty in English-to-Japanese translations of self-report questionnaires has also been found in other studies. Reversed items on the Japanese version of the Aggression Questionnaire had weaker factor loadings and differences between RWI and PWI were greater in Japanese compared to Western participants (Nakano, 2001). Measures of depression have shown similar findings (Iwata et al., 1995), with Japanese participants showing a different response pattern in RWI compared to PWI. Furthermore, the use of both RWI and PWI items reduces reliability more in Japanese compared to Western samples (Moschis et al., 2013), further demonstrating that reverse-worded items may be a particular issue in English-to-Japanese questionnaire translations. We therefore conclude that our poor item-total correlations for the reversed items is not an artefact of statistical error or translational differences, and instead reflect a wider pattern in transcultural research meriting further examination.

Bearing these issues in mind, the AFQ still showed good convergent validity. J-AFQ scores significantly correlated with EQ scores, albeit at a lower strength than in Western samples (Williams et al., 2016), and negatively correlated with autistic traits. While our ability to generalize to clinical populations is limited by the lack of participants on the autism spectrum in this sample, this again shows that the AFQ may be particularly useful in autism research and screening. Moreover, this convergent validity demonstrates that the AFQ is a valid way to measure motor empathy and cognition in Japanese participants.

In contrast to our predictions, we found that Japanese participants scored significantly lower on total AFQ scores and all three subscales compared to the Western group. Other studies comparing self-reported empathy in East Asian and Western cultures have found mixed responses. For instance, while Western participants score higher on the empathic concern subscale of the IRI, East Asian participants score higher in personal distress (Cassels et al., 2010).

Similar ambiguous findings emerge in Theory of Mind (ToM) research. Previous research suggests that Japanese children begin to pass False Belief and other Theory of Mind tasks later than Western children (Naito and Koyama, 2006), but that this difference is much smaller when examining non-verbal compared to verbal false belief tasks (Aival-Naveh et al., 2019). Neuroimaging work also suggests that Japanese participants recruit less from ToM areas in mentalising tasks compared to Western controls (Koelkebeck et al., 2011). They suggest that this is due to the greater cultural salience of mentalising and interpreting non-verbal behavior in Japan—as Japanese participants are more consistently taught to communicate through non-verbal cues, Japanese participants do not need to recruit as much neural activation to perform these tasks as Western participants.

Our findings may also be influenced by cultural differences in self-efficacy. In a comparison of 25 countries, Japan had the lowest average self-reported self-efficacy scores (Scholz et al., 2002). As such, Japanese participants may be more prone to rating the self negatively. However, behavioral studies suggest this may be due to modesty, rather than actual differences in self-belief or ability. For instance, while 72% of students rate their academic performance as below average in normal conditions, when offered a monetary incentive for more accurate ratings, the majority of participants then rated their performance as *above* average (Yamagishi et al., 2012). This demonstrates that while self-effacement may be common in Japanese populations, this may reflect a cultural norm, rather than true skill or beliefs about the self. As non-verbal emotional communication skill is highly valued in Japan (Shimizu, 2001), the AFQ may be particularly prone to these modesty effects.

This modesty effect may similarly account for the Differential Item Functioning results. Significant effects emerged across all three subscales in the differential item functioning analysis, even while controlling for age and gender. These suggest that Japanese and UK participants showed qualitative differences in response styles to several items and that items lack cross-cultural equivalence, potentially due to cultural differences or issues in translation. Modesty effects may lead to Japanese participants rating themselves more negatively on items measuring more socially desirable traits. Further studies utilizing qualitative approaches, such as interviews, or bilingual surveys, may shed light on these differences.

We also found fewer effects of gender on AFQ scores in Japanese participants. This aligns with well-established findings that gender differences in personality traits are mediated by culture. American and European cultures show larger gender differences in psychological outcomes compared to East Asian cultures (Costa et al., 2001). Other self-report measures of empathy also show this effect (Melchers et al., 2015; Zhao et al., 2019). We thus argue that the lack of gender differences are unlikely to constitute an issue regarding the AFQ's validity.

It must be noted there was a skewed gender ratio between our Japanese and Western samples, likely due to subtly different recruitment methods. The Japanese sample was recruited through an online survey company, while the Western sample was recruited online through convenience sampling. The convenience sampling, in both cases, may also restrict the generalizability of the results. Additionally, the Japanese sample was offered a small monetary incentive while Western participants were not. Furthermore, while multiple people were involved in the translation process and pains were taken to ensure translation was as robust as possible, the translation did not follow more robust standard guidelines, such as those outlined by Beaton et al. (2000). Finally, the test-retest reliability of the J-AFQ remains unclear. Future research should attempt to more stringently control these variables, as well as examine the test-retest reliability of the measure.

## Conclusions

Our study validated the Japanese translation of the AFQ, finding satisfactory convergent validity and internal reliability once reverse-items were accounted for. The AFQ is a novel self-report measure of motor empathy and cognition, which has been shown to reliably discriminate between autistic and non-autistic groups (Williams and Cameron, 2017). In line with this, low AFQ scores were associated with greater autistic traits in our Japanese sample. As few self-report measures of motor empathy exist within the literature, and non-verbal communication plays an important role in socialization, the AFQ represents a useful tool for research. We recommend the 13-item J-AFQ for use in research with general Japanese populations, although further validation work is necessary before it is suitable for clinical use. In particular, it may be useful to further examine how acquiescence impacts reporting of reverse-worded items.

Furthermore, we found evidence that self-reported motor empathy is diminished in Japanese compared to Western samples. However, it remains unclear whether this reflects differences in actual ability or self-report tendency. As Japanese participants may be more prone to understating their abilities due to cultural norms on modesty (Yamagishi et al., 2012), these differences may reflect general cultural tendencies in self-report rather than differences in actual ability. Future research may benefit from administering the AFQ alongside incentives to encourage more accurate self-report, or with behavioral measures of motor empathy.

## Data Availability Statement

The raw data supporting the conclusions of this article will be made available by the authors, without undue reservation.

## Ethics Statement

The studies involving human participants were reviewed and approved by Ethics Committee of the Unit for Advanced Studies of the Human Mind, Kyoto University and University of Aberdeen Ethics Review Board for the College of Life Sciences and Medicine. The patients/participants provided their written informed consent to participate in this study.

## Author Contributions

WS, JW, and SY conceived of and designed the study, as well as contributed to translating the AFQ. Data was collected by WS. Analysis was conducted by CH, IC, and NS. First draft of the manuscript was written by CH, and sections were written by IC and NS. Figure was created by IC. All authors contributed to manuscript revision, read, and approved the submitted version.

## Conflict of Interest

The authors declare that the research was conducted in the absence of any commercial or financial relationships that could be construed as a potential conflict of interest.

## Publisher's Note

All claims expressed in this article are solely those of the authors and do not necessarily represent those of their affiliated organizations, or those of the publisher, the editors and the reviewers. Any product that may be evaluated in this article, or claim that may be made by its manufacturer, is not guaranteed or endorsed by the publisher.

## References

[B1] Abell F. Happé F. Frith Y. (2000). Do triangles play tricks? attribution of mental states to animated shapes in normal and abnormal development. Cogn. Dev. 15, 1–16. 10.1016/S0885-2014(00)00014-9

[B2] Aival-Naveh E. Rothschild-Yakar L. Kurman J. (2019). Keeping culture in mind: a systematic review and initial conceptualization of mentalizing from a cross-cultural perspective. Clinic. Psychol. Sci. Pract. 12300. 10.1111/cpsp.12300

[B3] Baron-Cohen S. (2005). 共感する女脳、システム化する男脳 [Sympathising female brains, systemising male brains] Transl. by Miyake M. in Tokyo, Japan: NHK Publishing.

[B4] Baron-Cohen S. Wheelwright S. (2004). The empathy quotient: an investigation of adults with asperger syndrome or high functioning autism, and normal sex differences. J. Autism Dev. Disord. 34, 163–175. 10.1023/B:JADD.0000022607.19833.00 15162935

[B5] Beaton D. E. Bombardier C. Guillemin F. Ferraz M. B. (2000). Guidelines for the process of cross-cultural adaptation of self-report measures. Spine 25, 3186–3191. 10.1097/00007632-200012150-00014 11124735

[B6] Bjornsdottir R. T. Tskhay K. O. Ishii K. Rule N. O. (2017). Cultural differences in perceiving and processing emotions: a holistic approach to person perception. Cult. Brain 5, 105–124. 10.1007/s40167-017-0053-z

[B7] Blair R. J. (2005). Responding to the emotions of others: dissociating forms of empathy through the study of typical and psychiatric populations. Conscious. Cogn. 14, 698–718. 10.1016/j.concog.2005.06.004 16157488

[B8] Cassels T. G. Chan S. Chung W. Birch S. A. J. (2010). The role of culture in affective empathy: cultural and bicultural differences. J. Cogn. Cult. 10, 309–326. 10.1163/156853710X53120316427760

[B9] Cortina J. M. (1993). What is coefficient alpha? an examination of theory and applications. J. Appl. Psychol. 78:98. 10.1037/0021-9010.78.1.98

[B10] Costa P. T. Terracciano A. McCrae R. R. (2001). Gender differences in personality traits across cultures: robust and surprising findings. J. Pers. Soc. Psychol. 81, 322–331. 10.1037/0022-3514.81.2.322 11519935

[B11] Davis M. H. (1980). A multidimensional approach to individual differences in empathy. JSAS Catalog Select. Doc. Psychol. 10:85.

[B12] Decety J. Meyer M. (2008). From emotion resonance to empathic understanding: a social developmental neuroscience account. Dev. Psychopathol. 20, 1053–1080. 10.1017/S0954579408000503 18838031

[B13] Everitt B. S. (2002). The Cambridge dictionary of statistics (2nd ed.). Cambridge: Cambridge University press.

[B14] Hu L. T. Bentler P. M. (1999). Cutoff criteria for fit indexes in covariance structure analysis: conventional criteria versus new alternatives. Struct. Equ. Model. 6, 1–55. 10.1080/10705519909540118

[B15] Huggins C. F. Cameron I. M. Williams J. H. G. (2019). Different aspects of emotional awareness in relation to motor cognition and autism Traits. Front. Psychol. 10:2439. 10.3389/fpsyg.2019.02439 31749742 PMC6842938

[B16] Ishii K. Reyes J. A. Kitayama S. (2003). Spontaneous attention to word content versus emotional tone: differences among three cultures. Psychol. Sci. 14, 39–46. 10.1111/1467-9280.01416 12564752

[B17] Iwata N. Roberts C. R. Kawakami N. (1995). Japan-U.S. comparison of responses to depression scale items among adult workers. Psychiatry Res. 58, 237–245. 10.1016/0165-1781(95)02734-E 8570779

[B18] Johnson T. Kulesa P. Cho Y. I. Shavitt S. (2005). The relation between culture and response styles: evidence from 19 countries. J. Cross Cult. Psychol. 36, 264–277. 10.1177/0022022104272905

[B19] Khvatskaya Y. Lenzenweger M. F. (2016). Motor empathy in individuals with psychopathic traits: a preliminary study. J. Pers. Disord. 30, 613–632. 10.1521/pedi_2015_29_219 26168328

[B20] Koelkebeck K. Hirao K. Kawada R. Miyata J. Saze T. Ubukata S.. (2011). Transcultural differences in brain activation patterns during theory of mind (ToM) task performance in Japanese and Caucasian participants. Soc. Neurosci. 6, 615–626. 10.1080/17470919.2011.620763 21954949

[B21] Lebra T. S. (1976). Japanese Patterns of Behavior. Honolulu: University of Hawaii Press.

[B22] Lewis M. B. (2012). Exploring the positive and negative implications of facial feedback. Emotion 12:852. 10.1037/a0029275 22866886

[B23] Lord C. Risi S. Lambrecht L. Cook E. H. Leventhal B. L. DiLavore P. C.. (2000). The autism diagnostic observation schedule-generic: a standard measure of social and communication deficits associated with the spectrum of autism. J. Autism Dev. Disord. 30, 205–223. 10.1023/A:1005592401947 11055457

[B24] Masuda T. Nisbett R. E. (2001). Attending holistically versus analytically: comparing the context sensitivity of Japanese and Americans. J. Pers. Soc. Psychol. 81, 922–934. 10.1037/0022-3514.81.5.922 11708567

[B25] Matsumoto D. (1990). Cultural similarities and differences in display rules. Motiv. Emot. 14, 195–213. 10.1007/BF00995569

[B26] Matsumoto D. Yoo S. H. Fontaine J. Anguas-Wong A. M. Arriola M. Ataca B.. (2008). Mapping expression differences around the world: the relationship between emotional display rules and individualism versus collectivism. J. Cross Cult. Psychol. 39, 55–74. 10.1177/0022022107311854

[B27] Melchers M. Li M. Chen Y. Zhang W. Montag C. (2015). Low empathy is associated with problematic use of the Internet: empirical evidence from China and Germany. Asian J. Psychiatr. 17, 56–60. 10.1016/j.ajp.2015.06.019 26233696

[B28] Mori H. Mori K. (2013). An implicit assessment of the effect of artificial cheek raising: when your face smiles, the world looks nicer. Percept. Mot. Skills 116, 466–471. 10.2466/24.50.PMS.116.2.466-471 24032323

[B29] Moschis G. P. Ong F. S. Abessi M. Yamashita T. Mathur A. (2013). Cultural and sub-cultural differences in reliability: an empirical study in Japan and Malaysia. Asia Pacific J. Market. Logistics 25, 34–47. 10.1108/13555851311290920

[B30] Muncer S. J. Ling J. (2006). Psychometric analysis of the empathy quotient (eq) scale. Pers. Individ. Dif. 40, 1111–1119. 10.1016/j.paid.2005.09.020

[B31] Naito M. Koyama K. (2006). The development of false-belief understanding in Japanese children: delay and difference? Int. J. Behav. Develop. 30, 290–304. 10.1177/0165025406063622

[B32] Nakano K. (2001). Psychometric evaluation on the Japanese adaptation of the aggression questionnaire. Behav. Res. Ther. 39, 853–858. 10.1016/S0005-7967(00)00057-7 11419615

[B33] Rutter M. LeCouteur A. Lord C. (2003). The Autism Diagnostic Interview-Revised. Los Angeles, CA: Western Psychological Services.

[B34] Safdar S. Friedlmeier W. Matsumoto D. Yoo S. H. Kwantes C. T. Kakai H.. (2009). Variations of emotional display rules within and across cultures: a comparison between Canada, USA, and Japan. Can. J. Behav. Sci. 41, 1–10. 10.1037/a0014387

[B35] Sakai S. Wada N. Okuno H. Tatsumi A. Yammoto T. Yoshizaki A. (2014). Broad Autism Phenotype Questionnaire 日本語版 (BAPQ-J) J)の妥当性と信頼性の検討 [Broad Autism Phenotype Questionnaire—Japanese version: comparison between autism families and typically developing families] Jap. J.Clinic. Psychiatr. 43, 1181–1190.

[B36] Scholz U. Gutierrez Dona B. Sud S. Schwarzer R. (2002). Is general self-efficacy a universal construct? psychometric findings from 25 countries. Euro. J. Psychol. Assess. 18, 242–251. 10.1027//1015-5759.18.3.242

[B37] Schulte-Rüther M. Otte E. Adigüzel K. Firk C. Herpertz-Dahlmann B. Koch I.. (2017). Intact mirror mechanisms for automatic facial emotions in children and adolescents with autism spectrum disorder. Autism Res. 10, 298–310. 10.1002/aur.1654 27349835

[B38] Scott N. W. Fayers P. M. Aaronson N. K. Bottomley A. de Graeff A. Groenvold M.. (2010). Differential item functioning (DIF) analyses of health-related quality of life instruments using logistic regression. Health Qual. Life Outcomes 8:81. 10.1186/1477-7525-8-81 20684767 PMC2924271

[B39] Shimizu H. (2000). Japanese cultural psychology and empathic understanding: implications for academic and cultural psychology. Ethos 28, 224–247. 10.1525/eth.2000.28.2.224

[B40] Shimizu H. (2001). Japanese Adolescent Boys' Sense of Empathy (Omoiyari) and Carol Gilligans' perspectives on the morality of care: a phenomenological approach. Cult. Psychol. 7, 453–475. 10.1177/1354067X0174003

[B41] Steiger J. H. (2007). Understanding the limitations of global fit assessment in structural equation modelling. Pers. Individ. Dif. 42, 893–898. 10.1016/j.paid.2006.09.017

[B42] Strack F. Martin L. L. Stepper S. (1988). Inhibiting and facilitating conditions of the human smile: a nonobtrusive test of the facial feedback hypothesis. J. Pers. Soc. Psychol. 54:768. 10.1037/0022-3514.54.5.768 3379579

[B43] Tanaka A. Koizumi A. Imai H. Hiramatsu S. Hiramoto E. de Gelder B. (2010). I Feel Your voice: cultural differences in the multisensory perception of emotion. Psychol. Sci. 21, 1259–1262. 10.1177/0956797610380698 20713633

[B44] Van der Graaff J. Meeus W. de Wied M. van Boxtel A. van Lier P. A. C. Koot H. M.. (2016). Motor, affective and cognitive empathy in adolescence: interrelations between facial electromyography and self-reported trait and state measures. Cogn. Emot. 30, 745–761. 10.1080/02699931.2015.1027665 25864486

[B45] Van der Meer H. A. Sheftel-Simanova I. Kan C. C. Trujillo J. P. (2021). Translation, cross-cultural adaptation, and validation of a dutch version of the actions and feelings questionnaire in autistic and neurotypical adults. J. Autism Develop. Disorder. 5082. 10.1007/s10803-021-05082-w 34008098 PMC8938389

[B46] Williams J. H. G. Cameron I. M. (2017). The Actions and feelings questionnaire in autism and typically developed adults. J. Autism Dev. Disord. 47, 3418–3430. 10.1007/s10803-017-3244-8 28755033 PMC5633645

[B47] Williams J. H. G. Cameron I. M. Ross E. Braadbaart L. Waiter G. D. (2016). Perceiving and expressing feelings through actions in relation to individual differences in empathic traits: the Action and Feelings Questionnaire (AFQ). Cogn. Affect. Behav. Neurosci. 16, 248–260. 10.3758/s13415-015-0386-z 26486794 PMC4785213

[B48] Williams J. H. G. Whiten A. Singh T. (2004). A systematic review of action imitation in autistic spectrum disorder. J. Autism Dev. Disord. 34, 285–299. 10.1023/B:JADD.0000029551.56735.3a 15264497

[B49] Wong N. Rindfleisch A. Burroughs J. E. (2003). Do reverse-worded items confound measures in cross-cultural consumer research? the case of material value scale. J. Consum. Res. 30, 72–91. 10.1086/374697

[B50] Yamagishi T. Hashimoto H. Cook K. S. Kiyonari T. Shinada M. Mifune N.. (2012). Modesty in self-presentation: a comparison between the USA and Japan. Asian J. Soc. Psychol. 15, 60–68. 10.1111/j.1467-839X.2011.01362.x

[B51] Yoshie M. Sauter D. A. (2020). Cultural Norms Influence nonverbal emotion communication: japanese vocalizations of socially disengaging emotions. Emotion 20, 513–517. 10.1037/emo0000580 30816745

[B52] Zhao Q. Neumann D. L. Cao Y. Baron-Cohen S. Yan C. Chan R. C. K.. (2019). Culture-sex interaction and the self-report empathy in Australians and Mainland Chinese. Front. Psychol. 10:396. 10.3389/fpsyg.2019.00396 30914986 PMC6422933

[B53] Zieky M. (1993). Practical questions in the use of DIF statistics in test development, in Differential Item Functioning, eds Holland P. W. Wainter H. (Hillsdale, New Jersey: LawrenceErlbaum Associates), 337–348.

