# Supplementary material for: Cross-Cultural Differences and Psychometric Properties of the Japanese Actions and Feelings Questionnaire (J-AFQ)
Source: Front Psychol. 2021 Aug 20;12:722108. doi: 10.3389/fpsyg.2021.722108 (PMC8417608; doi:10.3389/fpsyg.2021.722108)
Supplement: Supplementary file 1 [file Data_Sheet_1.DOCX]

Supplementary Material

Japanese version of the Actions and Feelings Questionnaire.

自分自身について、最も当てはまると思う数字に丸をしてください。

|  |  | 全く当てはまらない | 少し当てはまらない | 少し当てはまる | 非常に当てはまる |
| --- | --- | --- | --- | --- | --- |
| 1 | 人のボディランゲージを読みがちである。 | 1 | 2 | 3 | 4 |
| 2 | 他者を理解するために、その人の表情やジェスチャーよりも言葉に頼る。 | 1 | 2 | 3 | 4 |
| 3 | 他者が何をしているかを理解するため、その人の行為の真似をするかもしれない。 | 1 | 2 | 3 | 4 |
| 4 | 好きな音楽を聞くと踊りたくなる。 | 1 | 2 | 3 | 4 |
| 5 | しばしば自分自身が何をしているかを心の目で見る。 | 1 | 2 | 3 | 4 |
| 6 | 電話で話しているとき、声のトーンで他者の感情を敏感に察知する。 | 1 | 2 | 3 | 4 |
| 7 | 他者が踊っていたら、一緒に踊りたくなる。 | 1 | 2 | 3 | 4 |
| 8 | 私の体の動きは、私の感じ方を反映してはいないことが多い。 | 1 | 2 | 3 | 4 |
| 9 | 日常の行為を遂行している自分をよく想像する。 | 1 | 2 | 3 | 4 |
| 10 | 感情のコミュニケーションにおいて触覚を重要視する。 | 1 | 2 | 3 | 4 |
| 11 | 他者が私に何を言ったかを思い出すとき、そのときの表情を思い出すのは難しい。 | 1 | 2 | 3 | 4 |
| 12 | 他者が本当に感じていることを推測するために、その人が私をどう見ているかに頼る。 | 1 | 2 | 3 | 4 |
| 13 | 他者が何も言わないとすれば、その人が感じていることを理解するのは難しい。 | 1 | 2 | 3 | 4 |
| 14 | しゃべるときによく手を動かす。 | 1 | 2 | 3 | 4 |
| 15 | 会話に熱中するとジェスチャーを使う。 | 1 | 2 | 3 | 4 |
| 16 | 私が他者に何かを言ったのを思い出すとき、その人の顔がどんな風だったかを、簡単に思い出すことができる。 | 1 | 2 | 3 | 4 |
| 17 | 行為するふりをするとものごとを理解しやすくなることがある。 | 1 | 2 | 3 | 4 |
| 18 | 誰かのボディランゲージを見ることは、私にとって感情を判断するためのいい方法ではない。 | 1 | 2 | 3 | 4 |

| **Supplementary Table**  Back-translations of reversed items, with original English and Japanese for comparison. | | |
| --- | --- | --- |
| Original English Item | Japanese Item | Blinded Back-Translation |
| To understand someone I rely on their words rather than their expression or gesture | 他者を理解するために、その人の表情やジェスチャーよりも言葉に頼る | I rely on what a given person says rather than their facial expression or gestures in order to understand other people. |
| My body movements do not tend to reflect the way I feel | 私の体の動きは、私の感じ方を反映してはいないことが多い | My physical actions do not often reflect what I am feeling. |
| When I recall what someone said to me, I have to think hard to remember their facial expression at the time | 他者が私に何を言ったかを思い出すとき、そのときの表情を思い出すのは難しい | If I am trying to recall what someone told me, it is difficult for me to remember their facial expression |
| I wouldn’t tend to know what someone was feeling if they did not say | 他者が何も言わないとすれば、その人が感じていることを理解するのは難しい | It is difficult for me to understand how another person is feeling unless they say something about it. |
| Watching somebody’s body language is not a good way to judge their feelings | 誰かのボディランゲージを見ることは、私にとって感情を判断するためのいい方法ではない | Simply observing another person's body language is not a reliable way for me to tell how someone is feeling. |
